# Correlation of Cytokines with Parasitic Infections, Undernutrition and Micronutrient Deficiency among Schoolchildren in Rural Tanzania: A Cross-Sectional Study

**DOI:** 10.3390/nu15081916

**Published:** 2023-04-15

**Authors:** Emmanuel C. Mrimi, Marta S. Palmeirim, Elihaika G. Minja, Kurt Z. Long, Jennifer Keiser

**Affiliations:** 1 Swiss Tropical and Public Health Institute, 4123 Allschwil, Switzerland; 2 University of Basel, 4001 Basel, Switzerland; 3 Ifakara Health Institute, Morogoro P.O. Box 53, Tanzania

**Keywords:** immunity, parasitic infection, undernutrition, microtrient deficiency, schoolchildren

## Abstract

Malnutrition and parasitic infections are often interconnected in a vicious cycle. Malnutrition can lead to changes in immune response, which may affect cytokine concentrations and potentially increase susceptibility to infections. In turn, parasitic infections can exacerbate malnutrition by impairing nutrient absorption. This cross-sectional study aimed to explore this interplay. Schoolchildren aged 6–12 years living in rural Tanzania (*n* = 120) provided blood, stool and urine samples to determine the relationship between cytokine concentrations (interleukin 4 (IL-4), interferon gamma (IFNγ) and interleukin 17A (IL-17A)), parasitic infections, undernutrition and micronutrient deficiency adjusting for sex, age, inflammatory markers, socioeconomic status and school categories. All schoolchildren had a normal blood cell count. The concentration of IL-4 was significantly higher in schoolchildren diagnosed with stunting, *Schistosoma mansoni* infection, a high C-reactive protein concentration, nausea, poor housing and increasing age. The concentration of IFNγ was associated with *Plasmodium falciparum* and *Entamoeba histolytica/Entamoeba dispar/Entamoeba moshkovskii* infections, vitamin A deficiency, attending the most remote schools and low socioeconomic status. Our study confirms a potential relationship between cytokine concentrations and parasitic infections, malnutrition and low socioeconomic status. A better understanding of long-term effects of parasitic infections and malnutrition on the immune function could help in designing tailored and effective interventions.

## 1. Introduction

Parasitic infections caused by intestinal nematodes, trematodes and protozoa often impair the intestinal and immune function, and lead to diarrheal syndrome, malnutrition, cognitive impairment and, consequently, death, if left untreated [1,2,3]. The majority of parasitic infections disproportionately affect poor people living in lower- and middle-income countries who are exposed to multiple biological, psychosocial and infrastructural risks [1,4,5]. Lack of access to basic healthcare services and education, adequate sanitation and clean water facilities, and malnutrition are among several risk factors that increase the susceptibility of individuals, especially children, to parasitic infections [6,7].

Malnutrition, such as undernutrition and micronutrient deficiency, plays a critical role in increasing the incidence and severity of malaria, intestinal nematodes and protozoa infections, through alteration of the structure and function of physical barriers (i.e., gut and skin) and impairment of the immune function [1]. Decreases in mucus production [8], megaloblastic anaemia [9] and limited production and activity of critical immune cell subsets [10], for example, are direct results of deficiencies of vitamin A, vitamin B12, folic acid and zinc, respectively. These deficiencies, in turn, increase the susceptibility of affected individuals to parasitic infections. Parasitic infections further exacerbate malnutrition by increasing nutrient loss, anorexia and demand for nutrients, and impair immune function, thus creating an endless vicious cycle of malnutrition and infection [1,2,3].

Infection with parasites triggers a series of immune reactions aiming at neutralizing the pathogen through both cell-mediated and humoral immune responses. Intracellular protozoan infections, such as *Entamoeba* spp. and *Plasmodium* spp., trigger a cell-mediated T helper 1 (Th1)-type immune response [11,12], which involves the activation and recruitment of phagocytic cells (macrophages and neutrophils) and secretion of inflammatory cytokines, such as interferon gamma (IFNγ) and interleukin 2, to the site of infection. In contrast, extracellular infections with metazoan parasites, such as nematodes and trematodes, can trigger a cell-mediated T helper 2 (Th2)-type immune response [13,14], which favours the secretion of interleukin 4 (IL-4), interleukin 6 and interleukin 13 cytokines that, in turn, mediate the recruitment of eosinophils, mast cells and basophils.

There is a significant body of research exploring the association between parasitic infections and nutritional status. However, less is known about how the combined effect of undernutrition and micronutrient deficiency within households and communities that differ in demographic structure and socioeconomic status alters the immune function and increases the individual’s susceptibility to parasitic infections. In this context, we conducted a cross-sectional survey with schoolchildren living in rural south-eastern Tanzania. Our findings on the prevalence of parasitic infections, undernutrition, micronutrient deficiency and demography have been published elsewhere [15,16]. The aim of the current study was to correlate the concentration of cytokines IL-4, IFNγ and interleukin 17A (IL-17A) with: (i) the presence or absence of parasitic infections, clinical signs and symptoms, undernutrition, anaemia, micronutrient deficiency, inflammatory and anaemia markers, and (ii) household and community risk factors such as drinking water sources and sanitation, housing quality, location of the school and socioeconomic status, which play a role in parasite transmission and infection.

## 2. Material and Methods

### 2.1. Ethics Statement

This cross-sectional study was granted ethical clearance from the National Institute of Medical Research (NIMR; Reference No. NIMR/HQ/R.8a/Vol. IX/3030), the Institutional Review Board of the Ifakara Health Institute (IRB-IHI; Reference No. IHI/IRB/No: 017-2018) in the United Republic of Tanzania, and the Ethikkommission Nordwest and Zentralschweiz (EKNZ; Reference No. 2018-00823) in Switzerland. All caregivers of children who were selected as potential participants were invited for an information session at the study school. Those who attended were informed about the study objectives, procedures, benefits and potential risks, and were encouraged to clarify any questions they may have. Caregivers who wanted their child to participate in the study were asked to sign an informed consent form. Those who could not read were asked to provide a thumbprint, and an impartial witness who could read and write signed the consent form, confirming all the relevant information was adequately conveyed to the caregiver. Each child was then invited to provide an oral assent.

### 2.2. Study Setting and Design

This secondary analysis utilised the data collected during a cross-sectional study that took place in Lungongole, Katrin, Kapolo, Kibaoni, Kilama A, Kilama B, Kikwawila, Milola and Site primary schools in the Kilombero district, Tanzania [15]. Data and analyses of blood (anaemia, micronutrient deficiency, and inflammatory and anaemia markers), stool and urine (parasitic infections) samples have been provided in previous publications [15,16]. Briefly, a total of 427 schoolchildren aged between 6 and 12 years old were randomly selected and consented to provide blood samples, which were centrifuged to collect the serum [16]. Of these, a subsample of 120 schoolchildren was selected for the measurement of cytokine concentrations. The selection of participants’ samples was performed as follows: one child with lymphatic filariasis, three infected with *Strongyloides stercoralis*, eight infected with soil-transmitted helminths, nine infected with *Plasmodium* spp., 24 infected with *Entamoeba histolytica/Entamoeba dispar/Entamoeba moshkovskii* infections, 24 infected with *Schistosoma mansoni* and 51 with no known parasitic infection.

### 2.3. Data and Sample Collection

Detailed information on the procedures for collecting stool and urine samples, anthropometric measurements, conducting physical and clinical examinations of participants and administering a structured questionnaire on socioeconomic status are described elsewhere [15,16]. Each participant provided a venous blood sample (about 7 mL) from the antecubital arm vein through an intravenous catheter into serum vacutainer tubes (BD Vacutainer^®^) and 1 mL in a tube containing Na2-EDTA (Sigma, St. Louis, MO, USA). Schoolchildren were not asked to fast or eat before sample collection. Blood samples were cold-chain-transported to the Ifakara Health Institute laboratory where, within six hours of collection, they were centrifuged to obtain serum. Serum samples were stored at −20 °C until they were shipped to Basel, Switzerland, for cytokine analysis.

### 2.4. Determination of Complete Blood Count and Cytokine Concentrations

The 1 mL of whole blood collected into the Na2-EDTA tubes was used for a complete blood count analysis of each sample using a Sysmex XP-300 Haematology Analyser (Sysmex Corporation, Kobe, Japan). The complete blood count analysis included the examination of white blood cells, red blood cells, lymphocytes and neutrophils. The analysis of the serum samples stored in vacutainer tubes was performed by the Rothen Laboratory, Basel, Switzerland. The detection of IL-4, IFNγ and IL-17A was performed using Multiplex U-PLEX equipment following a standard protocol, and the individual cytokine concentrations (in pg/mL) were then interpolated from the standard curve. Individual cytokine concentrations were later compared with their respective standard curves, and the cytokine readings falling outside the lower and higher detection limits were discarded. Concerning inflammatory markers, C-reactive protein (CRP) concentrations were determined using a Beckman coulter analyser, using anti-human CRP antibody with 0.2 mg/L as a quantification limit. Alpha (1)-acid glycoprotein (AGP) concentrations were measured using the sandwich ELISA method, as described by Erhardt et al. [17]. Data and analyses of haemoglobin and micronutrients’ concentrations have been presented in detail by Palmeirim et al. [15] and Mrimi et al. [16].

### 2.5. Statistical Analysis

All statistical analyses were conducted using R version 4.1.3 (R Core Team; R Foundation for Statistical Computing; Vienna, Austria). Descriptive distributions for complete blood count, inflammatory markers and cytokines stratified by sex, age group and location of schools were calculated. Complete blood counts and inflammatory markers were presented as the mean and standard deviation (SD), while cytokines were presented as the median and interquartile range, as they were not normally distributed.

Following the description by Palmeirim et al. [15] and Mrimi et al. [16], we categorised age, primary schools’ location and house quality into two categories each. Children aged 6 to 10 and 11 to 12 were categorised as younger and older children, respectively. Primary schools closer to and farther from the nearest town were categorised as the least rural schools and the most rural schools, respectively. The participants’ house quality was divided into better quality or poorer quality based on the building materials of the roof, walls and floor.

Inter-comparisons of stratified complete blood count were performed using a Student’s *t*-test, while stratified cytokine concentrations were compared using a Mann–Whitney U test. We created categorical variables for parasitic infections (*S. mansoni*, *Plasmodium falciparum* and *E. histolytica/E. dispar/E. moshkovskii*), clinical signs and symptoms (headache, abdominal pain and nausea), undernutrition (stunted, underweight and wasted), micronutrient deficiency (vitamin A, vitamin B12 and retinol-binding protein), anaemia, inflammatory and anaemia markers. In addition, we created categorical variables for household and community risk factors such as socioeconomic status, location of the primary schools, housing quality, shoe-wearing habits, drinking water sources, latrine ownership and its location. 

In a regression analysis, we explored the relationship between cytokine concentrations and primary risk factors, such as parasitic infections, clinical signs and symptoms, undernutrition, micronutrient deficiency, anaemia, inflammatory and anaemia markers. In addition, we looked at the influence of household and community risk factors such as socioeconomic status (least poor, poor and poorest), shoe-wearing habits, school categories (least vs. most rural schools), housing quality, drinking water sources, latrine ownership and its location on cytokine concentration in combination with the presence or absence of parasitic infection, clinical signs and symptoms, undernutrition and micronutrient deficiency.

Interleukin 4 and IFNγ concentrations were log-transformed prior to inclusion in the linear regression analysis. A multivariable log-linear regression model analysing associations between cytokines and primary risk factors, household risk factors and community risk factors was developed using an automated stepwise backward elimination procedure based on Akaike information criterion (AIC) to drop models with the highest AIC values. The final model was adjusted for sex, age, AGP, CRP, school categories and socioeconomic status. Both crude and adjusted coefficients generated from the log-linear model were reported. All results were considered significant at the level of *p* < 0.05.

## 3. Results

### 3.1. Parasitic Infection, Anthropometric Measurements, Nutritional Indicators, Clinical Signs and Symptoms, Complete Blood Counts, Inflammatory Markers and Cytokines Stratified by Sex, Age Groups and Location of the School

Among the 120 serum samples selected for cytokine analysis, 113 were successfully analysed. Table 1 summarises parasitic infections, anthropometric measurements, nutritional indicators, clinical signs and symptoms, complete blood count, inflammatory markers and cytokine concentrations stratified into sex, age groups and school categories of this cohort. Using a Chi-square test, we observed a higher mean white blood cell concentration of 5.84 cells/μL in boys, compared to 5.22 cells/μL in girls (*p* = 0.03). We also observed a significantly higher mean white blood cell concentrations of 5.80 cells/μL in younger children, compared to 5.13 cells/μL in older children (*p* = 0.02). We observed a similar phenomenon in neutrophils concentrations, where younger children had a higher mean concentration of neutrophils of 2.62 cells/μL, compared to 2.16 cells/μL in older children. Schoolchildren attending the most rural schools had significantly higher concentrations of lymphocytes, 2.68 cells/μL, compared to 2.37 cells/μL for children who attend the least rural schools (*p* = 0.02). For the case of nutritional indicators, we found that a higher proportion of older children (13.51 %) were diagnosed with stunting compared to younger children (11.71%; *p* = 0.04). In addition, a higher proportion of schoolchildren who attended the most rural schools (20.72%) were diagnosed with stunting compared to those attending the least rural schools (4.51%) (*p* = 0.003).

The median concentrations of IL-4 and IFNγ cytokines were 2.02 pg/mL and 7.23 pg/mL, respectively. Applying a Mann–Whitney analysis, we observed a significantly lower median IFNγ concentration of 5.79 pg/mL in children attending the most rural schools, compared to 9.29 pg/mL for those attending the least rural schools (*p* < 0.001; Table 1). We also observed a significantly higher median IL-4 concentration of 2.20 pg/mL in younger children, compared to 1.69 pg/mL in older children (*p* = 0.01). No significant difference in the median concentrations of either cytokine was observed between boys and girls. Results of IL-17A concentrations were below the detection limit, and hence they were not included in this analysis.

For inflammatory markers, 8 and 11 children among the 113 participants had elevated concentrations of CRP and AGP, respectively (Table 1). All children had normal concentrations of complete blood count. We did not find an association between parasitic infections, clinical signs and symptoms with the three strata (sex, age groups and school categories; Table 1). In addition, parasitic infections alone were not significantly associated with changes in complete blood counts, inflammatory markers or cytokine concentrations (Table 2).

### 3.2. Association of Cytokine Concentrations with Parasitic Infections, Clinical Signs and Symptoms

Using a regression analysis, we found higher mean concentrations of IL-4 (*p* = 0.01) among children infected with *S. mansoni* (Table 3). Mean IFNγ concentrations were significantly more elevated (*p* = 0.01) in children infected with *P. falciparum* (Table 4). Children diagnosed with a *E. histolytica/E. dispar/E. moshkovskii* infection had significantly lower mean IFNγ concentrations (*p* = 0.039) compared to those with no infection (Table 4). Children reporting nausea had significantly higher concentrations of IL-4 (*p* < 0.001) compared to those who did not report nausea (Table 3).

### 3.3. Association of Cytokine Concentrations with Undernutrition and Micronutrient Deficiency

Only IL-4 concentrations were associated with undernutrition. As summarised in Table 3, higher concentrations of IL-4 were observed among stunted schoolchildren (*p* = 0.02), as well as in schoolchildren diagnosed with higher concentrations of CRP in their blood (*p* = 0.02). Concerning micronutrient deficiencies, as shown in Table 4, schoolchildren diagnosed with vitamin A deficiency had a significantly higher concentration of IFNγ compared to their counterparts (*p* = 0.03). We did not find a significant association between anaemia and cytokine concentrations.

### 3.4. Association between Cytokine Concentrations and Other Risk Factors

When considering household and community factors, we found that children from the most remote schools had significantly lower mean IFNγ concentrations compared to their counterparts from the least remote schools (*p* = 0.01; Table 4). In addition, lower mean IFNγ concentrations were also found in children who were categorised into poor categories of the socioeconomic index (*p* = 0.03). For IL-4, we found that children living in houses with poorer quality were diagnosed with higher mean concentrations of IL-4 compared to their counterparts living in better-quality houses (*p* = 0.02; Table 3). Finally, we observed a negative relationship between age and IL-4 concentrations (*p* < 0.001).

## 4. Discussion

The current study has shed light on previously unexplored associations of cytokine concentrations (IL-4 and IFNγ, in particular) with parasitic infections, undernutrition, micronutrient deficiency, anaemia, clinical signs and symptoms, inflammatory markers and anaemia markers, as well as differences in risk factors such as socioeconomic status, location of the primary schools, shoe-wearing habit, housing quality, drinking water sources, latrine ownership and its location.

### 4.1. Association of Cytokine Concentrations with Parasitic Infections, Clinical Signs and Symptoms

We found elevated cytokine concentrations among schoolchildren who were infected with parasites, which is in line with previous studies [18,19]. The higher concentrations of IL-4 we observed in children diagnosed with *S. mansoni* infections are consistent with several previous publications [20,21,22]. Schistosome and soil-transmitted helminth infections are characterised by the secretion and release of IL-4, a Th2-immune response cytokine, which helps dislodge and expel parasites from the gut by stimulating a “weep and sweep” mechanism [23]. When schistosome infections are acute, there can also be a peak of a Th1-type immune response, followed by a switch to a Th2-type immune response later [22]. The association found between the higher mean concentrations of IL-4 and nausea might be due to the underlying intestinal helminth infections. Nausea, vomiting and abdominal pain, coupled with an elevation of a Th2-response, might occur in individuals harbouring intestinal helminth and schistosome infections [24,25].

In the case of IFNγ, we found higher mean concentrations in children infected with *P. falciparum*, which has been reported in several other studies [26,27,28]. Protozoa infections, such as *P. falciparum*, are known to trigger a strong Th1-type immune response, which is responsible for IFNγ secretion and release. To eliminate Plasmodium-infected red blood cells (merozoites), Th1-cells produce large amounts of IFNγ, which activate phagocytic cells that eliminate merozoites via phagocytosis. However, for other protozoa, such as *Entamoeba* spp., their link with IFNγ might not be direct, as we observed in this study. The unexpectedly low mean IFNγ concentration in schoolchildren diagnosed with *E. histolytica/E. dispar/E. moshkovskii* infection might be due to downregulation of IFNγ production caused by parasitic activation of an antagonistic Th2-type immune response [29,30,31,32]. This would be in line with what Guo and colleagues found through a murine experiment that demonstrated how *E. histolytica* evades the immune system through the downregulation of the Th1-type immune response that produces IFNγ [29]. *Entamoeba histolytica* achieves this feat by successfully activating the Th2-type immune response, flooding the system with anti-inflammatory cytokines such as IL-4 and IL10 and, in turn, suppressing the Th1-type immune response. 

### 4.2. Association of Cytokine Concentrations with Undernutrition, Micronutrient Deficiency and Inflammatory Markers

A combination of protein, carbohydrate and fat, and micronutrients such as vitamin A, vitamin B12, zinc, folate and vitamin D uptake, are vital sources of energy and significantly influence a person’s health [33,34]. Several studies have outlined the impact of malnutrition, such as undernutrition and micronutrient deficiency, on the immune response, showing that malnourished people are unable to mount a sufficient immune response against infections, especially a T-cell-mediated immune response [30]. Excess energy is required for the activation and sustenance of an immune response during infections, and the lack thereof results in an insufficient immune response to ward off infections, which can further worsen the condition by reducing nutrient uptake [31]. Rodríguez and colleagues observed that an energy-intensive Th1-type immune response, which is responsible for IFNγ secretion, is more likely to be impaired when a person is malnourished, due to insufficient energy and/or upregulation of the Th2-type immune response [32].

In the current study, we found an increased mean of IL-4 concentrations in stunted schoolchildren, which is in agreement with previous reports [32,35,36]. The immune response in malnourished children has been shown to be geared towards Th2 activation, resulting in the proliferation of anti-inflammatory cytokines, such as IL-4 and IL-10, which can increase the predisposition to protozoa and bacterial infections [32,35,37]. 

For CRP, schoolchildren diagnosed with elevated CRP concentrations were more likely to have higher concentrations of IL-4, which might be an indicator for inflammation associated with infections. C-reactive protein is a product of an acute-phase response during infection, responsible for clearing the invading pathogens [38]. To eliminate pathogens, CRP activates the classical complement pathway and enhances the complement pathogen-opsonisation process via binding to the pathogen polysaccharides [38]. In addition, CRP enhances the secretion of IL-4 during an acute-phase response by stimulating the expression of monocyte chemoattractant protein-1, which, in turn, increases the Th2-type immune response.

The higher mean concentrations of IFNγ associated with vitamin A deficiency found in this study are in line with what has been previously reported elsewhere [39,40]. Vitamin A deficiency is known to trigger a strong Th1-regulated IFNγ response, along with the downregulation of the Th2-response, which further predisposes individuals to protozoa and helminth infections.

### 4.3. Association between Cytokine Concentrations and Other Risk Factors

Poor communities are often vulnerable to infections due to the lack of disease prevention and healthcare services, adequate sanitation, safe drinking water and health education. Persistent chronic debilitating conditions can further exacerbate poverty. In the current study, a significant number of children living in poor housing had elevated concentrations of IL-4. On the other hand, lower mean concentrations of IFNγ were found in schoolchildren attending the most rural schools and in those classified into the poorer socioeconomic status category. In our previous publication [15], we showed that schoolchildren categorised into a poor socioeconomic status had a higher likelihood of being diagnosed with at least one parasitic infection, especially protozoa. Protozoa infections are one of the many factors that can lead to the downregulation of IFNγ, a Th1-type immune response cytokine, through the activation of the antagonist Th2-type immune response [13,22]. Using an unadjusted model, schoolchildren classified into the poorest socioeconomic status category were diagnosed with decreased concentrations of IFNγ (Table 4). However, those diagnosed with elevated CRP concentrations were more likely to have an increased concentration of IFNγ (Table 4).

The results from our linear regression analysis indicated that the older the child, the lower the IL-4 concentrations. An increase in age has been shown to be correlated with a reduction in intestinal parasitic infections [41,42], which subsequently leads to a lower activation and secretion of the anti-helminthic cytokine IL-4. Although not significant, in this study, a lower proportion of older schoolchildren were diagnosed with parasitic infections in comparison to younger schoolchildren. In the unadjusted model, schoolchildren diagnosed with low ferritin concentrations were more likely to have increased concentrations of IL-4.

### 4.4. Parasitic Infections, Clinical Signs and Symptoms, Complete Blood Counts and Inflammatory Markers 

The significant differences in mean concentrations of neutrophils and white blood cells observed between younger vs. older children, and children attending the most rural vs. the least rural schools, were deemed not clinically significant, since all study participants’ complete blood counts were within the normal range. Similar associations between the schoolchildren’s age groups, the location of the schools they attended and stunting were summarised by Mrimi et al. [16]. The higher proportion of schoolchildren diagnosed with elevated concentrations of AGP might indicate the presence of underlying/undiagnosed infections, inflammations and/or traumas. As an acute-phase protein, the concentration of AGP can increase by two- to five-fold in response to infections, inflammations and/or traumas [43].

### 4.5. Limitations

Firstly, due to the low prevalence of parasitic infections in our cross-sectional survey, the size of the subsample used in this study was relatively small. Second, a single time-point measure of cytokine concentrations in serum might not accurately inform the ongoing host–parasite interaction, and subsequently, the different antigenic stimulations during infection. Third, due to financial constraints, we could only analyse three types of cytokines; exploring other cytokines, such as interleukin 6, interleukin 10and tumor necrosis factor α, could strengthen our understanding of the immune response to parasitic infections and malnutrition. Finally, we did not analyse samples for the presence of bacterial infections, which are strong predictors of the Th1-response.

## 5. Conclusions

Our study has revealed potential relationships between cytokine concentrations, parasitic infections, malnutrition and their associated risk factors. Parasitic infections and malnutrition triggered highly polarised Th1- and Th2-responses in affected schoolchildren, which if not controlled, could lead to an increase in inflammation-related morbidity and susceptibility to infection. However, the immune mechanisms associated with malnutrition and parasitic infections, as well as their potential non-immediate and chronic effects, remain largely underexplored. This calls for longitudinal studies to better understand the impact of undernourishment on susceptibility to infections, and vice versa.

## Figures and Tables

**Table 1:** Parasitic infection, clinical signs and symptoms, complete blood counts and cytokine concentrations in schoolchildren in the Kilombero district, Tanzania.

	Girls	Boys	*p*-Value	6–10 Years Children	11–12 Years Children	*p*-Value	Most Rural Schools	Least Rural Schools	*p*-Value
Total number of children	53	60		71	41		65	48	
**Parasitic infection ^a^**									
Infected, n	34	41	0.64	48	27	0.85	47	28	0.12
Not infected, n	19	19		23	14		18	20	
**Anthropometric measurements**									
Height (cm), mean ± SD	131.06 ± 11.41	126.83 ± 9.06		124.14 ± 8.39	136.78 ± 8.52		127.51 ± 9.11	130.65 ± 11.84	
Weight (kg), mean ± SD	26.84 ± 6.52	126.83 ± 4.40		23.04 ± 3.58	30.45 ± 5.25		25.75 ± 4.90	25.75 ± 6.41	
**Nutritional indicators**									
Stunted, n (%)	13 (11.71)	15 (13.51)	0.96	13 (11.71)	15 (13.51)	**0.04**	23 (20.72)	5 (4.51)	**0.003**
Wasted, n (%)	9 (8.11)	12 (10.81)	0.68	13 (11.71)	8 (7.21)	0.90	9 (8.11)	12 (10.81)	0.11
Underweight, n (%)	7 (9.86)	6 (8.45)	0.41	13 (18.31)	0	*	9 (12.68)	4 (5.63)	0.35
**Clinical signs and symptoms ^b^**									
Headache, n									
Yes	7	8	0.98	9	6	0.77	58	40	0.36
No	46	52		62	35		7	8	
Abdominal pain, n									
Yes	15	17	1.00	19	13	0.58	16	32	0.31
No	38	43		52	28		49	16	
Nausea, n									
Yes	7	2	0.113 ^c^	4	5	0.219	7	2	0.35 ^c^
No	46	58		67	36		58	46	
**Complete blood count (cells/μL)**									
WBC, mean ± SD (×10^3^)	5.22 ± 1.62	5.84 ± 1.36	**0.03**	5.80 ± 1.59	5.13 ± 1.30	**0.02**	5.74 ± 1.38	5.28 ± 1.66	0.12
RBC, mean ± SD (×10^6^)	4.84 ± 0.74	4.87 ± 0.61	0.80	4.80 ± 0.56	4.98 ± 0.82	0.20	4.8 ± 0.50	4.93 ± 0.85	0.37
Lymphocytes, mean ± SD (×10^3^)	2.49 ± 0.64	2.6 ± 0.74	0.38	2.62 ± 0.74	2.43 ± 0.62	0.15	2.68 ± 0.68	2.37 ± 0.69	**0.02**
Neutrophils, mean ± SD (×10^3^)	2.25 ± 1.17	2.61 ± 0.94	0.07	2.62 ± 1.14	2.16 ± 0.87	**0.02**	2.51 ± 1.01	2.35 ± 1.14	0.43
**Inflammatory markers (mg/L)**									
CRP, median (IQR)	0.95 (0.50–1.93)	0.95 (0.50–2.70)	0.53	0.85 (0.50–2.33)	1.00 (0.60–2.30)	0.28	1.00 (0.50–2.30)	0.80 (0.50–2.43)	0.59
AGP, median (IQR)	0.59 (0.46–0.74)	0.63 (0.47–0.87)	0.38	0.59 (0.46–0.87)	0.61 (0.48–0.82)	0.99	0.61 (0.47–0.89)	0.59 (0.47–0.77)	0.39
**Cytokines (pg/mL)**									
IFNγ, median (IQR)	7.26 (4.56–19.14)	7.18 (5.41–11.63)	0.43	7.23 (4.88–12.33)	6.79 (4.72–12.03)	0.60	5.79 (4.22–8.57)	9.29 (6.71–20.68)	**<0.001**
IL-4, median (IQR)	2.20 (1.52–3.02)	1.92 (1.27–2.82)	0.30	2.20 (1.48–3.13)	1.69 (1.13–2.36)	**0.01**	2.02 (1.29–2.82)	2.07 (1.49–3.03)	0.60

Note: AGP, alpha (1)-acid glycoprotein; CRP, C-reactive protein; IFNγ, interferon gamma; IL-4, interleukin 4; IQR, interquartile range; RBC, red blood cells; SD, standard deviation; WBC, white blood cells. ^a^ Infection with at least *Entamoeba histolytica*/*Entamoeba dispar*/*Entamoeba moshkovskii, Plasmodium falciparum* or *Schistosoma mansoni*. ^b^ Chi-square test. ^c^ Chi-square test with Yates correction. * Zero outcome.

**Table 2:** Complete blood counts, inflammatory markers and cytokine concentrations in schoolchildren stratified by parasitic infections.

Variables	Parasitic Infection ^a^		*p*-Value
	Not Infected Children	Infected Children	
**Complete blood counts (cells/μL)**			
WBC, mean ± SD (×10^3^)	5.54 ± 1.45	5.55 ± 1.55	0.97
RBC, mean ± SD (×10^6^)	4.98 ± 0.88	4.80 ± 0.53	0.24
Lymphocytes, mean ± SD (×10^3^)	2.50 ± 0.60	2.58 ± 0.75	0.55
Neutrophils, mean ± SD (×10^3^)	2.52 ± 1.13	2.40 ± 1.04	0.61
**Inflammatory markers (mg/L)**			
CRP, median (IQR)	0.65 (0.65–2.51)	0.485 (0.22–1.20)	
High CRP concentration (%)	2 (2.41)	6 (7.23)	0.82 ^b^
AGP, median (IQR)	0.61 (0.50–0.85)	0.60 (0.46–0.82)	
High AGP concentration (%)	3 (3.13)	8 (8.33)	0.85 ^b^
**Cytokines (pg/mL)**			
IL-4, median (IQR)	2.01 (1.45–2.63)	2.09 (2.09–3.03)	0.71
IFNγ, median (IQR)	6.88 (4.38–12.38)	7.28 (4.86–11.94)	0.69

Note: AGP, alpha (1)-acid glycoprotein; CRP, C-reactive protein; IFNγ, interferon gamma; IL-4, interleukin 4; IQR, interquartile range; RBC, red blood cells; SD, standard deviation; WBC, white blood cells. ^a^ Infection with at least *Entamoeba histolytica*/*Entamoeba dispar*/*Entamoeba moshkovskii*, *Plasmodium falciparum* or *Schistosoma mansoni*. ^b^ Chi-square test with Yates correction.

**Table 3:** A multivariable log-linear stepwise backward modelling for the association of interleukin 4 with potential explanatory variables.

Variables	Crude Coefficient (95% CI)	*p*-Value	Adjusted Coefficient (95% CI)	*p*-Value
Stunted	−0.06 (−0.28, 0.17)	0.60	0.25 (0.04, 0.47)	**0.02**
*S. mansoni* infection	−0.003 (−0.23, 0.23)	1.00	0.27 (0.07, 0.46)	**0.01**
Macrocytic anaemia	0.13 (−0.19, 0.45)	0.40	0.23 (−0.09, 0.54)	0.15
Microcytic anaemia	0.20 (−0.27, 0.67)	0.40	0.35 (−0.06, 0.76)	0.09
Low ferritin concentration	0.27 (0.03, 0.51)	**0.03**	0.23 (−0.02, 0.48)	0.07
Low transferrin Concentration	0.20 (−0.12, 0.52)	0.20	0.15 (−0.16, 0.45)	0.34
Vitamin B12 deficiency	−0.34 (−0.72, 0.05)	0.09	−0.28 (−0.64, 0.07)	0.11
Poor housing quality	0.02 (−0.20, 0.24)	0.90	0.25 (0.04, 0.47)	**0.02**
Nausea	0.18 (−0.20, 0.57)	0.40	0.74 (0.38, 1.10)	**<0.001**
Poorer SES—middle	−0.16 (−0.46,0.15)	0.30	−0.16 (−0.46, 0.15)	0.31
Poorest SES—poor	−0.02 (−0.32, 0.28)	0.90	−0.14 (−0.46, 0.18)	0.38
Male schoolchildren	−0.10 (−0.31, 0.11)	0.40	0.03 (−0.15, 0.22)	0.72
High AGP concentration	−0.04 (−0.35, 0.27)	0.80	−0.21 (−0.51, 0.09)	0.17
High CRP concentration	0.04 (−0.30, 0.39)	0.80	0.45 (0.07, 0.83)	**0.02**
Most remote school	−0.024 (−0.24, 0.19)	0.800	0.04 (−0.15, 0.24)	0.64
Age	−0.10 (−0.16, −0.05)	**<0.001**	−0.14 (−0.20, −0.08)	**<0.001**

Note: AGP, alpha (1)-acid glycoprotein; CRP, C-reactive protein; IFNγ, interferon gamma; IL-4, interleukin 4; SES, socioeconomic status; *S. mansoni*, *Schistosoma mansoni*.

**Table 4:** A multivariable log-linear stepwise backward modelling for the association of interferon gamma with potential explanatory variables.

Variables	Crude Coefficient (95% CI)	*p*-Value	Adjusted Coefficient (95% CI)	*p*-Value
*S. mansoni* infection	0.30 (−0.02, 0.62)	0.06	0.35 (−0.03, 0.72)	0.07
*P. falciparum* infection	0.17 (−0.26, 0.60)	0.40	0.64 (0.17, 1.10)	**0.01**
*E. histolytica/E. dispar/E. moshkovskii* infection	−0.32 (−0.62, −0.01)	**0.04**	−0.44 (−0.85, −0.02)	**0.04**
Macrocytic anaemia	0.28 (−0.16, 0.71)	0.20	0.54 (−0.05, 1.13)	0.07
Microcytic anaemia	0.47 (−0.18, 1.11)	0.20	0.40 (−0.42, 1.22)	0.33
At least one micronutrient deficiency	−0.19 (−0.61, 0.24)	0.40	−0.46 (−0.95, 0.03)	0.06
Vitamin A deficiency	0.29 (0.001, 0.58)	0.05	0.45 (0.06, 0.85)	**0.03**
No toilet facility	−0.04 (−0.49, 0.42)	0.90	0.43 (−0.23, 1.09)	0.20
Poorer socioeconomic status	−0.61 (−1.0, −0.20)	**0.004**	−0.67 (−1.25, −0.09)	**0.03**
Poorest socioeconomic status	−0.62 (−1.0, −0.22)	**0.003**	−0.38 (−0.95, 0.20)	0.20
Male schoolchildren	−0.11 (−0.41, 0.18)	0.40	−0.21 (−0.57, 0.15)	0.24
High AGP concentrations	0.17 (−0.33, 0.67)	0.50	0.46 (−0.10, 1.02)	0.10
High CRP concentrations	0.75 (0.17, 1.3)	**0.01**	0.37 (−0.34, 1.08)	0.30
Most remote schools	−0.60 (−0.88, −0.33)	**<0.001**	−0.51 (−0.88, −0.15)	**0.01**
Age	−0.07 (−0.16, 0.01)	0.10	0.01 (−0.11, 0.12)	0.92

Note: AGP, alpha (1)-acid glycoprotein; CRP, C-reactive protein; *E. histolytica*/*E. dispar*/*E. moshkovskii*, *Entamoeba histolytica*/*Entamoeba dispar*/*Entamoeba moshkovskii*; IFNγ, interferon gamma; IL-4, interleukin 4; *P. falciparum*, *Plasmodium falciparum*; SES, socioeconomic status; *S. mansoni*, Schistosoma mansoni.

## Data Availability

The data presented in this study are available on request from the corresponding author.

## References

[1] Guerrant R.L., DeBoer M.D., Moore S.R., Scharf R.J., Lima A.A.M. (2013). The impoverished gut—A triple burden of diarrhoea, stunting and chronic disease. Nat. Rev. Gastroenterol. Hepatol..

[2] Ehrhardt S., Burchard G.D., Mantel C., Cramer J.P., Kaiser S., Kubo M., Otchwemah R.N., Bienzle U., Mockenhaupt F.P. (2006). Malaria, anemia, and malnutrition in african children--defining intervention priorities. J. Infect. Dis..

[3] Stephenson L.S. (1994). Helminth parasites, a major factor in malnutrition. World Health Forum..

[4] Jensen S.K.G., Berens A.E., Nelson C.A. (2017). Effects of poverty on interacting biological systems underlying child development. Lancet Child Adolesc. Health.

[5] The World Bank (2022). Poverty Overview: Development News, Research, Data. http://www.worldbank.org/en/topic/poverty.

[6] Ngure F.M., Reid B.M., Humphrey J.H., Mbuya M.N., Pelto G., Stoltzfus R.J. (2014). Water, sanitation, and hygiene (WASH), environmental enteropathy, nutrition, and early child development: Making the links. Ann. N. Y. Acad. Sci..

[7] Campbell O.M., Benova L., Gon G., Afsana K., Cumming O. (2015). Getting the basic rights-the role of water, sanitation and hygiene in maternal and reproductive health: A conceptual framework. Trop. Med. Int. Health.

[8] Yang Y., Yuan Y., Tao Y., Wang W. (2011). Effects of vitamin A deficiency on mucosal immunity and response to intestinal infection in rats. Nutrition.

[9] Girdwood R.H. (1951). Vitamin B12 and folic acid in the megaloblastic anaemias. Edinb. Med. J..

[10] Dardenne M. (2002). Zinc and immune function. Eur. J. Clin. Nutr..

[11] Gurung P., Kanneganti T.D. (2016). Immune responses against protozoan parasites: A focus on the emerging role of Nod-like receptors. Cell. Mol. Life Sci..

[12] Velazquez C., Dominguez V., Garzon T., Rascon R. (2018). Immunity to Protozoa. eLS.

[13] McSorley H.J., Maizels R.M. (2012). Helminth infections and host immune regulation. Clin. Microbiol. Rev..

[14] Wynn T.A. (2015). Type 2 cytokines: Mechanisms and therapeutic strategies. Nat. Rev. Immunol..

[15] Palmeirim M.S., Mrimi E.C., Minja E.G., Samson A.J., Keiser J. (2021). A cross-sectional survey on parasitic infections in schoolchildren in a rural Tanzanian community. Acta Trop..

[16] Mrimi E.C., Palmeirim M.S., Minja E.G., Long K.Z., Keiser J. (2022). Malnutrition, anemia, micronutrient deficiency and parasitic infections among schoolchildren in rural Tanzania. PLoS Negl. Trop. Dis.

[17] Erhardt J.G., Estes J.E., Pfeiffer C.M., Biesalski H.K., Craft N.E. (2004). Combined measurement of ferritin, soluble transferrin receptor, retinol binding protein, and C-reactive protein by an inexpensive, sensitive, and simple sandwich enzyme-linked immunosorbent assay technique. J. Nutr..

[18] Ntigui C.N.M.M., Oyegue-Liabagui S.L., Kouna L.C., Imboumy K.R., Tegomo N.P.T., Okouga A.P., Ontoua S., Lekana-Douki J.-B. (2021). Inflammatory cytokine responses in children with asymptomatic malaria infection living in rural, semi-urban and urban areas in south-eastern Gabon. Clin. Exp. Immunol..

[19] Cox F.E., Liew E.Y. (1992). Centrefold: T-cell subsets and cytokines in parasitic infections. Parasitol. Today.

[20] Pearce E.J., Macdonald A.S. (2002). The immunobiology of schistosomiasis. Nat. Rev. Immunol..

[21] Fairfax K., Nascimento M., Huang S.C.-C., Everts B., Pearce E.J. (2012). Th2 responses in schistosomiasis. Semin. Immunopathol..

[22] Anthony R.M., Rutitzky L.I., Urban J.F., Stadecker M.J., Gause W.C. (2007). Protective immune mechanisms in helminth infection. Nat. Rev. Immunol..

[23] Harris N.L., Loke P. (2017). Recent Advances in Type-2-Cell-Mediated Immunity: Insights from Helminth Infection. Immunity.

[24] Jourdan P.M., Lamberton P.H.L., Fenwick A., Addiss D.G. (2018). Soil-transmitted helminth infections. Lancet.

[25] Bethony J., Brooker S., Albonico M., Geiger S.M., Loukas A., Diemert D., Hotez P.J. (2006). Soil-transmitted helminth infections: Ascariasis, trichuriasis, and hookworm. Lancet.

[26] Miller J.L., Sack B.K., Baldwin M., Vaughan A.M., Kappe S.H. (2014). Interferon-mediated innate immune responses against malaria parasite liver stages. Cell Rep..

[27] Schofield L., Villaquiran J., Ferreira A., Schellekens H., Nussenzweig R., Nussenzweig V. (1987). Gamma interferon, CD8+ T cells and antibodies required for immunity to malaria sporozoites. Nature.

[28] Ferreira A., Schofield L., Enea V., Schellekens H., van der Meide P., Collins W.E., Nussenzweig R.S., Nussenzweig V. (1986). Inhibition of development of exoerythrocytic forms of malaria parasites by gamma-interferon. Science.

[29] Guo X., Stroup S.E., Houpt E.R. (2008). Persistence of Entamoeba histolytica infection in CBA mice owes to intestinal IL-4 production and inhibition of protective IFN-gamma. Mucosal Immunol..

[30] Schaible U.E., Kaufmann S.H. (2007). Malnutrition and infection: Complex mechanisms and global impacts. PLoS Med..

[31] Pelletier D.L. (1994). The potentiating effects of malnutrition on child mortality: Epidemiologic evidence and policy implications. Nutr. Rev..

[32] Rodríguez L., González C., Flores L., Jiménez-Zamudio L., Graniel J., Ortiz-Muñiz R. (2005). Assessment by flow cytometry of cytokine production in malnourished children. Clin. Diagn. Lab. Immunol..

[33] Carreiro A.L., Dhillon J., Gordon S., Higgins K.A., Jacobs A.G., McArthur B.M., Redan B.W., Rivera R.L., Schmidt L.R., Mattes R.D. (2016). The Macronutrients, Appetite, and Energy Intake. Annu. Rev. Nutr..

[34] Yakoob M.Y., Lo C.W. (2017). Nutrition (Micronutrients) in Child Growth and Development: A Systematic Review on Current Evidence, Recommendations and Opportunities for Further Research. J. Dev. Behav. Pediatr..

[35] Rytter M.J., Kolte L., Briend A., Friis H., Christensen V.B. (2014). The immune system in children with malnutrition–A systematic review. PLoS ONE.

[36] González-Martínez H., Rodríguez L., Nájera O., Cruz D., Miliar A., Domínguez A., Sánchez F., Graniel J., González-Torres M.C. (2008). Expression of cytokine mRNA in lymphocytes of malnourished children. J. Clin. Immunol..

[37] Monk J.M., Steevels T.A.M., Hillyer L.M., Woodward B. (2011). Constitutive, but not Challenge-Induced, Interleukin-10 Production is Robust in Acute Pre-Pubescent Protein and Energy Deficits: New Support for the Tolerance Hypothesis of Malnutrition-Associated Immune Depression Based on Cytokine Production in vivo. Int. J. Environ. Res. Public Heal..

[38] Sproston N.R., Ashworth J.J. (2018). Role of C-Reactive Protein at Sites of Inflammation and Infection. Front. Immunol..

[39] Cantorna M.T., Nashold F.E., Hayes C.E. (1995). Vitamin A deficiency results in a priming environment conducive for Th1 cell development. Eur. J. Immunol..

[40] Ahmad S.M., Haskell M.J., Raqib R., Stephensen C.B. (2009). Markers of innate immune function are associated with vitamin a stores in men. J. Nutr..

[41] Hotez P.J., Bundy D.A., Beegle K., Brooker S., Drake L., de Silva N., Montresor A., Engels D., Jukes M., Chitsulo L. (2006). Helminth Infections: Soil-Transmitted Helminth Infections and Schistosomiasis, in Disease Control Priorities in Developing Countries.

[42] Bundy D.A.P., Cooper E.S., Thompson D.E., Didier J.M., Simmons I. (1988). Effect of age and initial infection intensity on the rate of reinfection with Trichuris trichiura after treatment. Parasitology.

[43] Hochepied T., Berger F.G., Baumann H., Libert C. (2003). Alpha(1)-acid glycoprotein: An acute phase protein with inflammatory and immunomodulating properties. Cytokine Growth Factor Rev..

